# Tackling cryptococcal meningitis in Nigeria, one-step at a time; the impact of training

**DOI:** 10.1371/journal.pone.0235577

**Published:** 2020-07-06

**Authors:** Rita O. Oladele, Alexander Jordan, Patrick Akande, Sulaimon A. Akanmu, Iorhen E. Akase, Sani Aliyu, David W. Denning, Tom Chiller

**Affiliations:** 1 College of Medicine, University of Lagos, Lagos, Nigeria; 2 Mycotic Diseases Branch, Centers for Disease Control and Prevention (CDC), Atlanta, Georgia, United States of America; 3 APIN Public Health Initiatives, Abuja, Nigeria; 4 National Agency for the Control of AIDS (NACA), Abuja, Nigeria; 5 Global Action Fund for Fungal Infections (GAFFI), Geneva, Switzerland; University of Minnesota, UNITED STATES

## Abstract

**Background:**

Nigeria is estimated to have 25,000 cases of cryptococcal antigenemia (CrAg) annually. CrAg screening with pre-emptive fluconazole treatment is recommended but not yet implemented in Nigeria. Trainings were conducted to improve health-care provider (HCP) awareness and clinical skills in the management and prevention of cryptococcal meningitis (CM).

**Methods:**

HCPs providing care for people living with HIV were targeted for training at 13 sites from April to November 2018 Course content was adapted from CDC Cryptococcal Screening Program Training Manual and LIFE-website. “Hands-on” training on CrAg testing and lumbar puncture was included. A 14-point pre and post-test assessment instrument was designed to capture the impact of the training and focus group discussions (FGDs) were conducted.

**Results:**

A total of 761 HCPs were trained. 519 HCPs completed the pre-test evaluation while 470 (90.6%) took part in the post-test evaluation. Post-training, HCPs were significantly more likely to respond correctly to all 14 assessment items, with the mean percentage score rising to 91.0% from a pre-training value of 60.0%. FGDs revealed that many of the HCPs were not aware of the CrAg screening and pre-emptive treatment recommendations in Nigerian guidelines, and reported not having seen or managed a case of CM. Also, they highlighted challenges with routine CrAg screening due to a lack of access to CD4 testing, CrAg test kits, antifungal drugs, as well as the need for similar trainings across all tiers of care in Nigeria.

**Conclusion:**

Training significantly improved HCPs’ understanding of Nigerian policy on CrAg screening, CM diagnosis and best management practices. This training could be included in routine capacity building efforts for HCPs involved in HIV care in Nigeria.

## Introduction

Cryptococcosis remains a substantial cause of morbidity and mortality among HIV-infected patients, especially in sub-Saharan Africa, where it is estimated to cause up to 15% of AIDS-related deaths [1]. A recently updated analysis of cryptococcal disease estimated a global incidence of 223,100 cases annually of which 73% occur in sub-Saharan Africa [2]. Furthermore, 75% of the estimated 181,100 deaths associated with cryptococcal disease occur in sub-Saharan Africa [1]. Point-of-care diagnostic assays have revolutionized the diagnosis of this deadly opportunistic infection, especially in areas with limited laboratory infrastructure [3]. Overt cryptococcal disease is known to be preceded by a period of asymptomatic cryptococcal antigenemia that can last days to months [2]. Screening for cryptococcal antigenemia (CrAg) coupled with pre-emptive antifungal therapy for CrAg-positive patients without overt cryptocococcal disease has been demonstrated as a cost-effective strategy with survival benefits [4]. Routine CrAg screening and treatment has been recommended by WHO since 2011 and has now been incorporated into HIV national guidelines in more than 2 dozen countries, including Nigeria [5, 6] However, routine CrAg screening is not yet routinely implemented in Nigeria.

Nigeria has a population of 190 million people and accounts for 9% of all people living with HIV worldwide, with an HIV prevalence of 1.4% amongst persons aged 15–49 [7]. The distribution of PLHIV in Nigeria varies markedly due to the wide disparity in HIV prevalence rates by state and geographical region; state-specific estimates range from 1.0% to 12.7% [8]. The recent global estimate of cryptococcal disease indicates that Nigeria has >25,000 cases of cryptococcosis annually [1]. A study from North-Central Nigeria revealed a 36% hospital-based frequency of cryptococcal meningitis, with high mortality [9].

The 2017 WHO Advanced HIV guidelines encourage early diagnosis of cryptococcal antigenemia using rapid cryptococcal antigen (CrAg) assays followed by pre-emptive fluconazole for CrAg-positive patients without cryptococcal meningitis [10]. The guidelines recommend amphotericin B and/or flucytosine (5-FC)-based regimens for CM treatment, a minimum package of toxicity prevention, monitoring and management for those receiving amphotericin B, and appropriate timing of ART initiation for those with cryptococcal meningitis. While the Nigeria HIV treatment guidelines reflect the WHO recommendations, routine implementation of these recommendations in Nigeria has proved to be a challenge. This may be partially due to poor awareness of national recommendations on the part of health care providers (HCPs) involved in the management of patients with advanced HIV disease. Additional challenges in Nigeria include poor uptake of lumbar punctures (LP) for both diagnostic and therapeutic purposes, as well as a lack of necessary equipment to perform screening, CM treatment, and treatment monitoring. For example, very few pharmacies in Nigeria currently stock amphotericin B (which has been licensed in Nigeria for the last 50 years) and 5-FC is not yet registered in Nigeria. Fluconazole is generally available, but often only at sub-optimal dosages (50mg or 100mg tabs), leading to a high pill burden for patients receiving pre-emptive fluconazole therapy.

To reduce CM-related mortality in PLHIV in Nigeria, a training curriculum was developed which aims to raise awareness and improve clinical skills of HCPs in the recognition, diagnosis, management, and prevention of HIV-associated cryptococcal meningitis. The primary goal of this training package was to increase the clinical index of suspicion amongst HCPs managing patients with advanced HIV disease and to stimulate policy review and effective implementation of current recommendations in Nigeria.

## Methods

This report is the result of training conducted with approval obtained from APIN Public Health Initiatives, National Agency for the Control of AIDS (NACA) and local hospital management. These organizations convened the trainings, while MMSN provided technical expertise for the trainings. Informed written consent was obtained from all participants and data was analysed anonymously. These organizations convened the trainings, while MMSN provided technical expertise for the trainings. Trainings took place from May to November 2018, with two sites undergoing training per month.

This was a mixed method study comprising of a pre- and post-test design (quantitative) and focus group discussion (qualitative). HCPs providing care for PLHIV in outpatient and inpatient settings of secondary and tertiary health facilities across the six geopolitical zones of Nigeria were targeted for the training program.

### Course syllabus

The training curriculum consisted of a 9-module course with the 9^th^ module consisting of a focus group discussion (FGD). This course content was adapted from the CDC Cryptococcal Screening Program Training Manual for Healthcare Providers (CS232487-A 08/2012) and course materials from the www.LIFE-Worldwide.org website. These materials were developed at the University of Manchester, in collaboration with Professor Tom Harrison’s cryptococcal meningitis research group at University of London. Each module had specific intended learning objectives. Modules included basic background information on CrAg and CM as well as interactive case studies. Practical ‘hands on’ sessions for CrAg testing and LP simulations were conducted using a practice mannequin (Lumbar Puncture Model; Limbs and Things®). Certificates of trainings were awarded to participants upon completion of the modules and post-test assessments.

Modules covered the following topics; ‘Overview of cryptococcal meningitis’, ‘What is Cryptococcus and cryptococcosis?’, ‘Recognizing signs and symptoms of cryptococcal disease’, ‘How to perform a lumbar puncture’, ‘Diagnosing cryptococcal disease’, ‘Treating cryptococcal meningitis’, ‘Antifungal drug interactions’, ‘Preventing cryptococcal meningitis’, ‘Decision-making guide for cryptococcal screening’ and ‘Your role as a health care provider’ and ‘The way forward (focus group discussions)’.

### Target audience

A total of 13 sites were selected (7 APIN-supported sites and 6 non-APIN sites). Training facilitators as well as “champions” were selected from each site before training in order to encourage greater site-ownership of the trainings. The primary targets of trainings were HIV clinic doctors, infectious disease clinicians, clinical microbiologists, intensivists, pediatricians and staff from other cadres which deal with immunocompromised patients on a routine basis. Other HCP cadres targeted included laboratory scientists, nurses, counselors, and pharmacists. They all had basic professional qualifications (for example, Registered Nurse) or bachelor’s degree in their fields while some also had higher qualifications, including master’s degree, PhD and professional fellowship (Medical Consultants). A total of at least 32 clinicians per site were requested to attend the training. Multiple FGD groups of eight HCPs each were created at each site. FGD participants were chosen systematically by random number selection at the end of the training. Moderators were responsible for leading the FGD (tasks included posing questions, stimulating discussion as needed, and giving suggestions to the focus group); moderators were also tasked with summarizing the key issues discussed and then presenting these issues to the full class at the end of the FGD. The main goal of the FGDs was to highlight challenges with diagnosis and treatment of CM in the Nigerian setting, identify gaps, and offer potential solutions going forward. Continuous Medical Education (CME) points were awarded at the end of training.

Two pack of test kits were left for each site so that CrAg screening for the ‘at risk’ population commenced immediately.

### Facilitators

The facilitators for this training included a clinical microbiologist, a neurologist (for demonstration of the performance of LP), an infectious disease specialist, and a pharmacist.

### Study instrument

The questionnaire used for the pre- and post-test assessment consisted of two sections and a total of 14 items. The first section included 8 items focused on the diagnosis of cryptococcal meningitis while the second section included 6 items focused on pre-emptive antifungal and CM treatment recommendations. The participants were asked to respond to each item statement with a “True (T)” or “False (F)” answer. Each correct response received a score of “1” while “0” was given for a wrong response. There were maximum possible points of 8 and 6 for the diagnosis and treatment sections respectively. All correct answers for items were decided by the training team before the training program and were based on current WHO and Nigerian HIV program recommendations. The exact phrasing of questions was decided based on what the training team felt the respondents would be most likely to understand.

### Data analysis

Quantitative data analysis was done using IBM SPSS Statistics version 24. The sociodemographic characteristics of participants were described with means and frequencies. The pre- and post-test scores of the respondents on the assessment instrument were calculated and analyzed as percentage scores (total score/14 x 100%). The mean percentage scores were then calculated, and paired t-tests were conducted to test the significance of the change in overall mean percentage scores from pre to post-test. McNemar tests were conducted to assess the significance of differences in the number of correct and incorrect responses to questionnaire items from pre to post-test. The significance level was set at 0.05. The findings from the FGDs were analyzed in line with identified themes.

## Results

A total of 761 participated in the training. Before the training, the assessment instrument was completed by 519 participants (pre-test) while 470 of them (90.6%) took the post-test, yielding an attrition rate of 9.4%.

The majority of participants completing the pre-test assessment were male (59.2%). Doctors comprised the majority (349 participants or 67.2%) of HCP participants; followed by nurses– 78 (15.0%), medical laboratory staff– 51 (9.8%), and pharmacists– 41 (7.9%). The mean age of the participants was 39.4 years while the mean years of practice or work experience was 12.0 years. One hundred and fifty (29%) reported having seen or managed a case of CM; with an average of 5 self-estimated cases seen, (median: 2, range: 1–100). Only 81 (15.6%) HCPs were aware of the current Nigerian recommendations on CrAg screening and pre-emptive treatment.

Before the training, 4 of 14 questions were correctly answered by over 80% of the participants (Table 1). Questions 3 and 14 had the fewest number of correct responses before the training (19.1% and 23.1% correct, respectively). Performance improved following the training, with more than 80% of respondents correctly responding to 12 of the 14 questions. More than 90% of respondents correctly answered 10 out of the 14 items at post-test. Item 3, which 19.1% of respondents correctly answered at pre-test improved to 82.8% correct on the post-test. The number of respondents who correctly answered all questions increased from 0.4% to 29.1% following the training. Furthermore, most of the patients (81.3%) correctly identified the eligible patients for CrAg screening based on CD4 count, and 75.5% of them correctly identified pre-emptive treatment as treatment of patients with serological or imaging evidence of infection even when clinical disease is absent.

**Table 1:** Number and proportion of participants with correct responses.

	Statement	Pre-test with correct responses (N = 519), n (%)	Post-test with correct responses (N = 470), n (%)	Difference in proportions with correct responses
	**Diagnosis**			
**1**	The following clinical features are suggestive of cryptococcal meningitis in HIV-infected patients: headache, neck rigidity, and vomiting (*True*)	424 (81.7)	470 (100.0)	18.3
**2**	There is a need to screen both ART-experienced and ART-naïve HIV-infected patients with CD4+ cell count <100cells/mm3 for prior subclinical cryptococcal disease (*True*)	422 (81.3)	454 (96.6)	15.3
**3**	All HIV-infected patients regardless of CD4+ cell count level should be screened (*False*)	99 (19.1)	389 (82.8)	63.7
**4**	Cryptococcal meningitis can be diagnosed in the laboratory in less than 1 hour (*True*)	305 (58.8)	453 (96.4)	37.6
**5**	A blood sample as simple as a needle prick can be used to diagnose cryptococcal antigenemia (CrAg) (*True*)	302 (58.2)	462 (98.3)	40.1
**6**	Only CSF samples can be used to diagnose cryptococcal meningitis (*False*)	268 (51.6)	355 (75.5)	23.9
**7**	CrAg lateral flow assay (LFA) can also be used as a CSF test for cryptococcal meningitis (*True*)	318 (61.3)	438 (93.2)	31.9
**8**	A positive cryptococcal antigenemia test is a cause for concern for the managing clinician (*True*)	421 (81.1)	450 (95.7)	14.6
	**Treatment**			
**9**	It is cheaper to do screening and pre-emptive therapy than definitive management (*True*)	424 (81.7)	449 (95.5)	13.8
**10**	Pre-emptive treatment is defined as treatment of patients who have serological or imaging evidence of infection but clinical disease is yet to develop (*True*)	392 (75.5)	442 (94.0)	18.5
**11**	Definitive therapy (treating the disease after onset of symptoms and signs) for cryptococcal meningitis is more cost effective than pre-emptive therapy (*False*)	238 (45.9)	349 (74.3)	28.4
**12**	There is no challenge of drug–drug interaction of antifungals and ARTs (*False*)	301 (58.0)	423 (90.0)	32.0
**13**	Therapeutic lumbar puncture improves outcomes in management of cryptococcal meningitis (*True*)	349 (67.2)	446 (94.9)	27.7
**14**	Steroids are needed in management of cryptococcal meningitis (*False*)	120 (23.1)	406 (86.4)	63.3
	**Total number and proportion with all responses correct**	**2 (0.4)**	**137 (29.1)**	**28.7**

McNemar significance testing showed that the proportions of respondents responding correctly was significantly higher for all questions following the training (see Table 2).

**Table 2:** Changes in responses following training.

	Question	Pre-test/Post-test					
1	The following clinical features are suggestive of cryptococcal meningitis in HIV-infected patients: headache, neck rigidity, and vomiting (True)		**Post-test**				
		**Pre-test**		Correct	Incorrect	**Total**	p-value
			Correct	388	0	**388**	<0.001
			Incorrect	82	0	**82**	
			**Total**	**470**	**0**	**470**	
2	There is need to screen both ART-experienced and ART-naïve HIV-infected patients with CD4+ cell count <100cells/mm3 for prior subclinical cryptococcal disease (True)		**Post-test**				
		**Pre-test**		Correct	Incorrect	**Total**	p-value
			Correct	376	9	**385**	<0.001
			Incorrect	78	7	**85**	
			**Total**	**454**	**16**	**470**	
3	All HIV-infected patients regardless of CD4+ cell count level should be screened (False)		**Post-test**				
		**Pre-test**		Correct	Incorrect	**Total**	p-value
			Correct	70	18	**88**	<0.001
			Incorrect	319	63	**382**	
			**Total**	**389**	**81**	**470**	
4	Cryptococcal meningitis can be diagnosed in the laboratory in less than 1 hour (True)		**Post-test**				
		**Pre-test**		Correct	Incorrect	**Total**	p-value
			Correct	260	10	**270**	<0.001
			Incorrect	193	7	**200**	
			**Total**	**453**	**17**	**470**	
5	Blood sample as simple as a needle prick can be used to diagnose cryptococcal antigenemia (CrAg) (True)		**Post-test**				
		**Pre-test**		Correct	Incorrect	**Total**	p-value
			Correct	263	4	**267**	<0.001
			Incorrect	199	4	**203**	
			**Total**	**462**	**8**	**470**	
6	Only CSF samples can be used to diagnose cryptococcal meningitis (False)		**Post-test**				
		**Pre-test**		Correct	Incorrect	**Total**	p-value
			Correct	194	47	**241**	<0.001
			Incorrect	161	68	**229**	
			**Total**	**355**	**115**	**470**	
7	CrAg lateral flow Assay (LFA) can also be used as a CSF test for cryptococcal meningitis (True)		**Post-test**				
		**Pre-test**		Correct	Incorrect	**Total**	p-value
			Correct	267	16	**283**	<0.001
			Incorrect	171	16	**187**	
			**Total**	**438**	**32**	**470**	
8	A positive cryptococcal antigenemia test is a cause for concern for the managing clinician (True)		**Post-test**				
		**Pre-test**		Correct	Incorrect	**Total**	p-value
			Correct	367	15	**382**	<0.001
			Incorrect	83	5	**88**	
			**Total**	**450**	**20**	**470**	
9	It is cheaper to do screening and pre-emptive therapy than definitive management (True)			**Post-test**			
		**Pre-test**		Correct	Incorrect	**Total**	p-value
			Correct	369	14	**383**	<0.001
			Incorrect	80	7	**87**	
			**Total**	**449**	**21**	**470**	
10	Pre-emptive treatment is defined as treatment of patients who have serological or imaging evidence of infection but clinical disease is yet to develop (True)		**Post-test**				
		**Pre-test**		Correct	Incorrect	**Total**	p-value
			Correct	338	17	**355**	<0.001
			Incorrect	104	11	**115**	
			**Total**	**442**	**28**	**470**	
11	Definitive therapy (treating the disease after onset of symptoms and signs) for cryptococcal meningitis is more cost effective than pre-emptive therapy (False)		**Post-test**				
		**Pre-test**		Correct	Incorrect	**Total**	p-value
			Correct	189	24	**213**	<0.001
			Incorrect	160	97	**257**	
			**Total**	**349**	**121**	**470**	
12	There is no challenge of drug–drug interaction of antifungals and ARTs (False)		**Post-test**				
		**Pre-test**		Correct	Incorrect	**Total**	p-value
			Correct	250	17	**267**	<0.001
			Incorrect	173	30	**203**	
			**Total**	**423**	**47**	**470**	
13	Therapeutic lumbar puncture improves outcomes in management of cryptococcal meningitis (True)		**Post-test**				
		**Pre-test**		Correct	Incorrect	**Total**	p-value
			Correct	303	16	**319**	<0.001
			Incorrect	143	8	**151**	
				**446**	**24**	**470**	
14	Steroids are needed in management of cryptococcal meningitis (False)		**Post-test**				
		**Pre-test**		Correct	Incorrect	**Total**	p-value
			Correct	95	10	**105**	<0.001
			Incorrect	311	54	**365**	
				**406**	**64**	**470**	

The post-test mean percentage scores for all categories of HCPs were significantly higher following the training, as shown in Table 3. At pre-test, doctors had the highest mean score, followed by medical laboratory personnel. After the training, the doctors still had the highest mean percentage scores, followed by pharmacists. Pharmacists had the highest percentage increase in scores, followed by nurses. Overall, the post-test mean percentage score of the respondents rose from a pre-training value of 60.0% to 91.0% post-training (p<0.001).

**Table 3:** Mean percentage scores of participant by work category.

Category of HCP	Pre-test mean percentage score, % (SD)	Post-test mean percentage score, % (SD)	Difference in pre- and post-test mean percentage scores, %	% Increase in mean percentage score	p-value
Doctors (n = 308)	61.6 (17.5)	92.5 (8.17)	30.9	50.2	<0.001
Medical laboratory staff (n = 50)	60.0 (15.3)	88.1 (8.11)	28.1	46.8	<0.001
Pharmacists (n = 39)	57.1 (17.2)	89.4 (10.2)	32.3	56.6	<0.001
Nurses (n = 73)	54.4 (16.0)	82.2 (7.98)	27.8	51.1	<0.001
**Total (n = 470)**	**60.0 (17.2)**	**91.0 (8.58)**	**31.0**	**51.7**	**<0.001**

### Focus group discussions

A total of 85 focus group discussions were held, ranging from 4–10 per site within the training period. Each discussion group presented a summary of their discussion to the other trainees and staff following the discussion period.

Regarding awareness of current WHO or Nigerian national recommendations for diagnosis and pre-emptive treatment of cryptococcosis, low levels were exhibited by most of the FGD participants and the majority of them were not aware that the Nigerian HIV program already recommended routine CrAg screening and pre-emptive fluconazole to prevent CM. According to one participant:

“We did not know that WHO and our National guidelines recommended routine CrAg screening pre-emptive fluconazole use in HIV-infected patients with advanced disease”

Their response included a lack of awareness that diagnosis of CM in symptomatic PLHIV can be promptly carried out using whole-blood antigen testing in the absence of LP and CSF testing, as shown by this statement

“This is the first time I am hearing of such a test, to imagine that it can help save a life.”

Similarly, there was poor knowledge of procedures and complications of CM treatment in HIV-infected patients, as reported here:

“This training is an eye-opener, I routinely would give any patient with suspected raised intracranial pressure–steroids and I was not aware of possible drug-drug interactions. The app shared is extremely useful.”

Asked whether they had seen and managed cases of CM previously, most of the participants responded in the negative. One of them said:

“All I think of is TB meningitis because of the high rate of TB in our setting.”

Common concerns and points raised in the discussions included the following:

- CD4 counts (which are needed to target CrAg screening) are not always available (due to lack of reagents and poor machine maintenance).
- There is a lack of availability of and accessibility to antifungal drugs and CrAg diagnostics (the CrAg LFA was not licensed in Nigeria at the time of the trainings).
- Challenges exist with regards to funding of routine CrAg screening, as it is not presently included in the routine package of care for PLHIV in Nigeria.
- There is a lack of comprehensive data on the true burden of the ‘at risk’ (PLHIV with advanced HIV disease) population in Nigeria which would enable relevant implementing partners to strategize for routine implementation.

Focus group discussions also discussed next steps and the “way forward”, some general consensuses from respondents across the sites included:

- Trainings should be prepared for community health extension workers (CHEWs) to enable identification of patients at risk and in need of CrAg screening at the lower tiers of care.
- Step-down trainings should be prepared and provided for all ART facilities in Nigeria
- Job aids based on Nigerian recommendations need to be designed to guide clinicians in implementation of CrAg screening and CM treatment
- Educational aids should be developed for patients with advanced HIV disease and CrAg/CM
- There is a crucial need to improve access to amphotericin B in Nigeria
- More must be done to encourage clinicians to routinely do LPs in patients that need it, both for diagnosis and as therapy in managing raised intracranial pressure.

## Discussion

The results of this training program highlight some of the challenges with diagnosis, treatment, and management of cryptococcal meningitis (CM) among PLHIV in Nigeria. Some sites had uniquely challenging situations including rapid turnover of doctors providing care for HIV patients. This has led to many doctors lacking formal training in HIV medicine, with only basic knowledge of HIV-related complications. Before the training, respondents generally showed poor understanding of the danger posed by undiagnosed cryptococcosis.

The result of the FGDs revealed that the participants exhibited low levels of awareness about current Nigerian recommendations on diagnosis and pre-emptive treatment of cryptococcosis, with the majority of respondents not aware that the Nigerian HIV program already recommends routine CrAg screening and pre-emptive fluconazole to prevent CM. Respondents also displayed low levels of knowledge on the procedures and complications of CM treatment in HIV-infected patients, including the potential for drug-drug interactions and current recommendations to avoid using steroids during the induction phase of CM treatment. The failure in the wide dissemination of current Nigerian CM/CrAg guidance among clinicians constitutes an important barrier to providing optimal care to HIV patients. In addition, no CrAg LFA tests were licensed or available in Nigeria at the time of the training. Although the IMMY CrAg LFA^©^ is registered in Nigeria as of 2019, routine procurement of the test has not yet occurred. Therefore, there is currently no feasible method to screen at-risk patients. The implementation of CrAg screening among HIV-infected patients elsewhere has been shown to improve outcomes among HIV-infected patients and has reduced the incidence of cryptococcosis induced IRIS in newly diagnosed HIV-infected patients [11, 12].

Most of the participants claimed that they had neither seen nor managed a case of cryptococcal meningitis previously. However, a recent study showed that cryptococcal meningitis accounted for 36% of all suspected cases of meningitis among HIV-infected patients in Jos (North-Central Nigeria) [9]. Nigeria has been estimated to have a large burden of both asymptomatic CrAg (>25,000) and overt CM (27,100) in 2014 [1]. Therefore, the low proportion of clinicians reporting having previously managed cases of CM could be due in part to a low level of awareness, low index of suspicion, and a lack of diagnostic capacity that has likely led to CM going undiagnosed and untreated.

Another hallmark of the pre-training assessment was the low level of knowledge on current WHO and Nigerian recommendations for the diagnosis of cryptococcosis. There was a particular lack of awareness that diagnosis of CM in symptomatic PLHIV (in the absence of LP and CSF testing) can be done in minutes using whole-blood antigen testing, which can greatly expedite CrAg and CM diagnosis in both in-patient and out-patient settings. Another important knowledge gap was that most clinicians believed that steroids should be given to patients diagnosed with CM for the treatment of raised intracranial pressure (ICP), which goes against current WHO and Nigerian recommendations. Many clinicians reported giving empiric steroids for the control of raised intracranial pressure (ICP) in the setting of sub-acute meningitis, as many cases are assumed to be caused by mycobacterium tuberculosis until proven otherwise. However, the use of corticosteroids during the induction phase of CM treatment is associated with an increased occurrence of adverse events and disability outcomes at 6 weeks, and is therefore not recommended for use in CM patients [13]. Knowledge of the central role of lumbar puncture in PLHIV, for diagnosis of CM and for therapeutic reduction of raised ICP was very low before training. Use of therapeutic lumbar punctures in the early stages of treatment is central to the successful treatment of CM, as receipt of LP during CM treatment has been shown to significantly reduce mortality [14].

The FGDs highlighted several common challenges that currently impede the successful implementation of CrAg screening and treatment in Nigeria. One common challenge discussed was the lack of availability of CD4 counts for risk stratification of newly diagnosed HIV-infected patients. The revised WHO guidelines on the management of cryptococcosis advocates for the screening of all HIV-infected patients with a CD4+ T-cell count of ≤ 100 cell/mm^3^, although a cut-off of ≤ 200 cell/mm^3^ may be considered. In many health care facilities in Nigeria, the results for CD4 cell counts only become available weeks after samples are taken. This makes practical application of the WHO recommended CrAg screening strategy difficult in the context of rapid ART initiation for newly diagnosed PLHIV. Due to the delay in CD4 results, patients are generally placed on ART before the CD4 result (and any associated diagnostic results, such as CrAg) become available to the HCP. This means that severely immunocompromised patients who are at risk for CM or who have asymptomatic or pauci-symptomatic disseminated cryptococcal disease and/or CM may be at increased risk of IRIS due to ART-initiation before receiving appropriate antifungal treatment.

The knowledge gained from this training may lead to increased case detection of cryptococcosis, including CM. Such a scenario will raise awareness of another major challenge, namely the non-availability and inaccessibility of antifungals to treat cryptococcosis in Nigeria. Amphotericin B and flucytosine are recommended for induction phase of treatment. Although amphotericin B has been licensed in Nigeria for the past 50 years, it is available in only two pharmacies in Nigeria and is often prohibitively expensive; flucytosine is not licensed in Nigeria and is currently unavailable.

The benefits of this training were shown by the marked improvement in the post-training assessment of the knowledge on the diagnosis and treatment of cryptococcosis in the setting of advanced HIV. By November 2019, 250 cases of CM had been captured in the national HIV disease database, compared to 0 cases the year before. Additionally, the training helped identify several access issues currently limiting widespread implementation of routine CrAg screening in Nigeria, including unavailability of CrAg kits, unavailability of antifungal agents, and unavailability of timely CD4 counts in Nigeria. By generating awareness and discussion around these issues, this training program has helped generate demand for said commodities on the part of HCPs and ultimately lead to improved forecasting and increased procurement on the part of policy makers and implementing partners in Nigeria. As a result of advocacy generated by this training, the Federal Ministry of Health (FMoH) has constituted an AHD national task team, which has adopted the training curriculum.

In conclusion, there is a need to improve access to commodities needed for routine CrAg screening and appropriate CM treatment within the Nigerian HIV program, including fluconazole, CrAg test kits, amphotericin B, and flucytosine. New strategies to expedite the return of CD4 results to ART clinicians should be explored in order to appropriately integrate routine CrAg screening without compromising rapid ART initiation for newly diagnosed PLHIV in Nigeria. This training program demonstrated significant improvement in the knowledge of CrAg screening, CM treatment, and CM management on the part of Nigerian HCPs. The gains and momentum created at the hospital level by this training program would be further consolidated through facilitation of additional step-down trainings at the peripheral health-care facilities.

## Supporting information

S1 Data (XLSX)
